# Drug Use Evaluation of Direct Oral Anticoagulants (DOACs) in Patients With Advanced Cirrhosis

**DOI:** 10.7759/cureus.24029

**Published:** 2022-04-11

**Authors:** Lindsey Jarboe, Apaar Dadlani, Sudeepthi Bandikatla, Regan Wade, Ashutosh Barve

**Affiliations:** 1 Pharmacy, University of Louisville Hospital, Louisville, USA; 2 Internal Medicine, University of Louisville, Louisville, USA; 3 Gastroenterology, Hepatology, and Nutrition/Internal Medicine, University of Louisville, Louisville, USA

**Keywords:** vte, doac, thromboembolism, rivaroxaban, apixaban, anticoagulants, cirrhosis

## Abstract

Objectives: Low molecular weight heparin and vitamin K antagonists are commonly used in cirrhotic patients requiring anticoagulation. However, their monitoring with anti-factor Xa and international normalized ratio (INR) may not be reliable in cirrhosis. Direct oral anticoagulants (DOACs) do not need laboratory monitoring, making these agents a favorable alternative. However, apixaban and rivaroxaban have been avoided in advanced liver disease due to their metabolism in the liver. The purpose of this medication use evaluation was to assess the use of DOACs, specifically apixaban and rivaroxaban, in patients with cirrhosis.

Methods: We performed a retrospective, single-center study. Inpatients who had a diagnosis of cirrhosis and received at least one dose of a DOAC (apixaban or rivaroxaban) from April 2016 through June 2020 at our hospital were included in the analysis. Data collected included the reason for admission, Child-Pugh classification, renal function, if this was a home medication or newly started as an inpatient medication, indication, and dosing. The clinical efficacy outcome (new venous thromboembolic event (VTE) or progression of old VTE), and clinical safety outcome (bleeding event) were analyzed.

Results: 41 patients with cirrhosis were treated with apixaban or rivaroxaban. Based on the Child-Pugh classification, 29.3% (n=12/41) were placed on a DOAC outside of the FDA prescribing recommendations. In this subpopulation, 8.3% (n=1/12) patients had venous thromboembolism (VTE) and 16.6% (n=2/12) had bleeding events. Overall, 7.3% patients (n=3/41) had VTE and 4.8% (n=2/41) had bleeding events. In the Apixaban for the Initial Management of Pulmonary Embolism and Deep-Vein Thrombosis as First-Line Therapy (AMPLIFY) trial comparing the efficacy and safety profile of apixaban with enoxaparin/warfarin therapy in acute VTE, 2.3% of patients had VTE and 15% had bleeding events.

Conclusion: Our study demonstrated that it may be possible to safely use DOACs in patients with advanced cirrhosis. Further studies are needed to evaluate the safety and efficacy of DOACs in this patient population, as our study was limited by the small sample size and its retrospective design.

## Introduction

Vitamin K antagonists and low molecular weight heparin have been traditionally used for anticoagulation in patients with cirrhosis. Anti-factor Xa (anti-FXa) activity and synthesis of antithrombin is inversely related to the severity of liver disease [1], and the international normalized ratio (INR) is often prolonged in advanced cirrhosis, which limits the monitoring of these agents in this population. Direct oral anticoagulants (DOACs) do not need laboratory monitoring, making these agents a favorable alternative. However, apixaban and rivaroxaban have been avoided in advanced liver disease due to their metabolism in the liver. The purpose of this medication use evaluation was to assess the use of DOACs, specifically apixaban and rivaroxaban, which are direct FXa inhibitors, in patients with cirrhosis at our tertiary care hospital. Furthermore, the safety and efficacy outcomes of these agents when used in patients with cirrhosis will also be evaluated.

The abstract for this study was submitted to the American College of Gastroenterology (ACG) 2021 Annual Scientific Meeting & Postgraduate Course Conference, October 22-27, 2021, Las Vegas, Nevada, United States (Hybrid) [2].

## Materials and methods

We performed a retrospective, single-center study. Inpatients who had a diagnosis of cirrhosis and received at least one dose of a DOAC (apixaban or rivaroxaban) from April 2016 through June 2020 at the University of Louisville Hospital, Louisville, US, were included in the analysis. Patients receiving dabigatran were not included in this analysis due to it being a non-formulary DOAC agent at our hospital. We excluded patients with insufficient evidence of cirrhosis or patients who were prescribed prophylactic anticoagulation after surgeries. This information was obtained via a report from pharmacy informatics, which included patient demographics and dates of first administration. The University of Louisville Institutional Review Board approved the study (IRB protocol number: 21.1008).

Data collected included the reason for admission, Child-Pugh classification, renal function, if this was a home medication or newly started as an inpatient medication, indication, and dosing. In our study, the Child-Pugh classification was used for hepatic dysfunction categorization according to the Food and Drug Administration (FDA) product labeling recommendations for each DOAC. The clinical efficacy outcome (new venous thromboembolic event (VTE) or progression of old VTE), and clinical safety outcome (bleeding event) were analyzed for the initial as well as any subsequent hospital admissions after the DOAC was initiated. A bleeding event was classified as (i) minor if it did not necessitate any dose adjustments or drug discontinuation, and no intervention was required, (ii) moderate, if the event necessitated intervention and/or medication adjustment but was not life-threatening, and (iii) major, if the event was potentially fatal. A thrombotic event was defined as a new VTE or progression of VTE. 

## Results

We found a total of 41 patients with cirrhosis who were placed on a DOAC (apixaban or rivaroxaban) between April 2016 and June 2020. Baseline characteristics of included patients are listed in Table 1.

**Table 1:** Patient demographics SD: standard deviation

Patient Demographics	
Age, years (mean ± SD)	61.6 ± 10.9
Gender	Male: 60.4%, Female: 39.6%
Race	Caucasian: 76.7%, African American: 23.3%, Other: 0%
Body Mass Index (mean ± SD)	29.4 ± 7.70
Child-Pugh Score (mean ± SD)	7.63 ± 1.74

**Figure 1:**
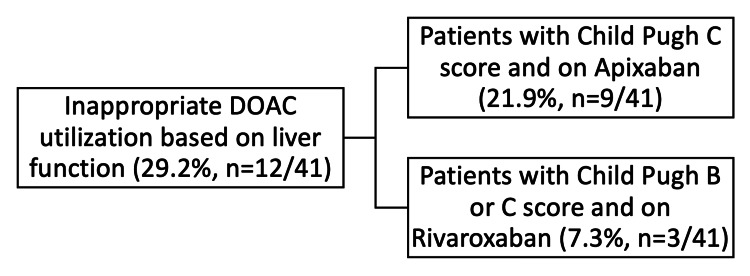
Inappropriate DOAC use based on liver function DOAC: direct oral anticoagulant

FDA product labeling recommends avoiding rivaroxaban in patients with Child-Pugh B and C cirrhosis, and apixaban in patients with Child-Pugh C cirrhosis. In our study cohort, 70.7% (n=29/41) patients had appropriate DOAC utilization based on the Child-Pugh classification while the remaining 29.3% (n=12/41) were on a DOAC outside of the FDA prescribing recommendations per the product labeling (Figure 1).

**Figure 2:**
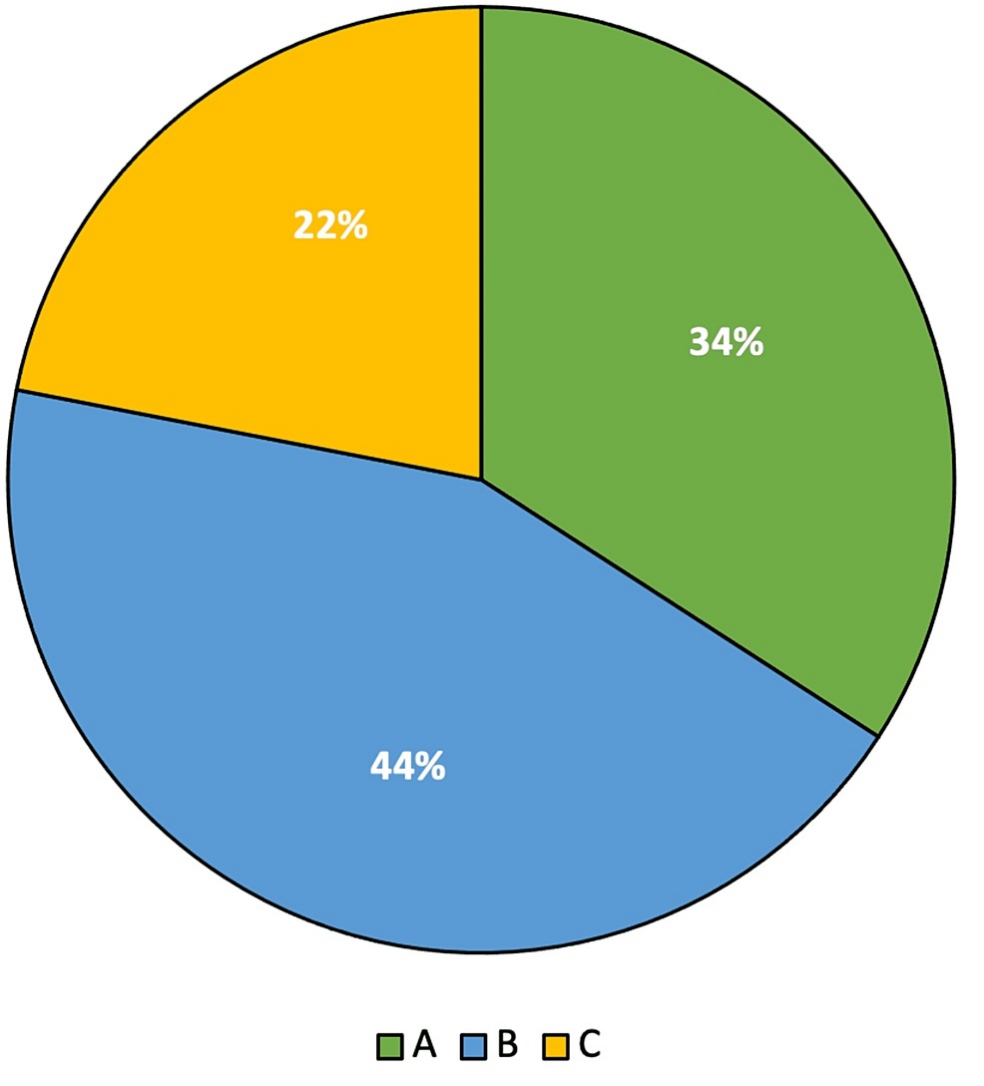
Child-Pugh Class

**Figure 3:**
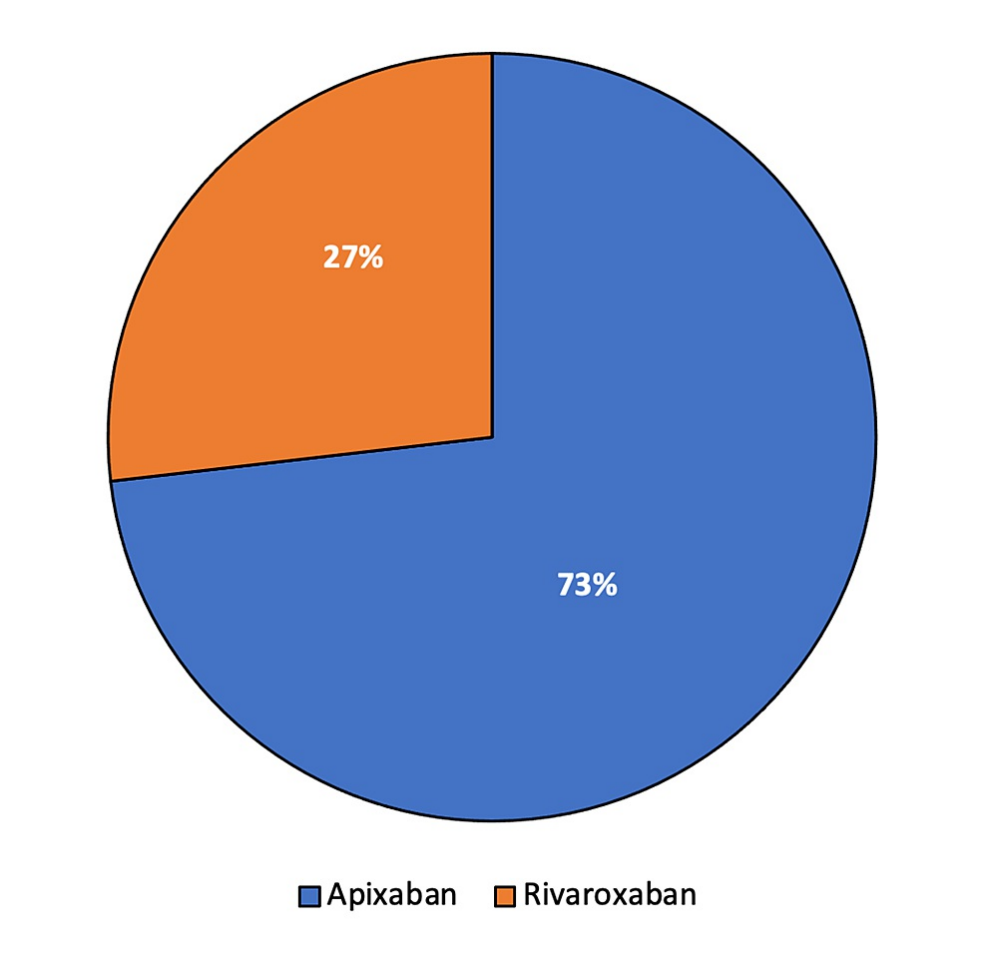
DOAC utilized DOAC: direct oral anticoagulant

Child-Pugh class, choice of DOAC agent utilized, and category of initiation are shown in Figure 2 and Figure 3, respectively.

**Figure 4:**
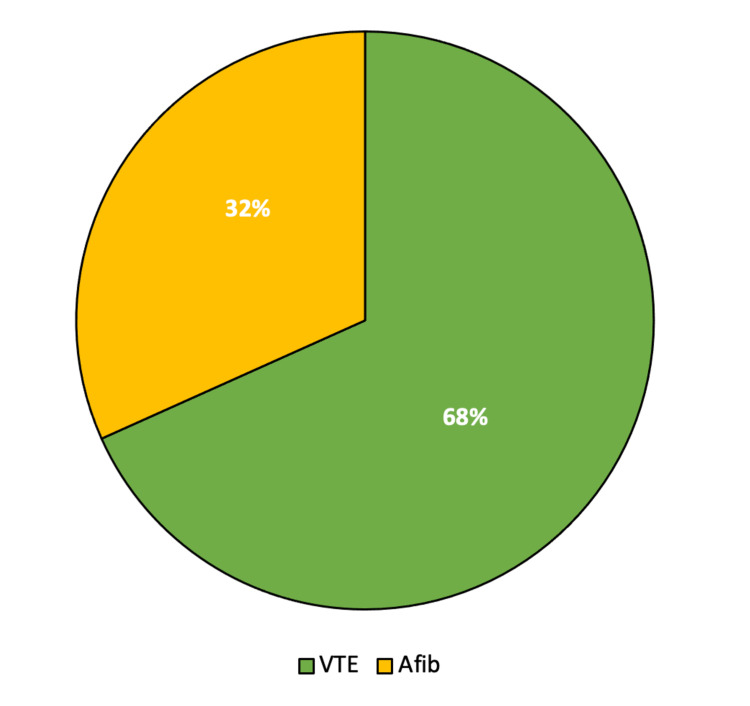
Indication for DOAC use VTE: venous thromboembolism; Afib: atrial fibrillation; DOAC: direct oral anticoagulant

The indications for anticoagulation (Figure 4) were atrial fibrillation or VTE, including portal vein thrombosis, deep vein thrombosis, and pulmonary embolism.

Patients that received a DOAC outside of package insert recommendations based on liver function are reviewed in Table 2.

**Table 2:** Inappropriate DOAC utilization based on liver function (29.2%, n=12/41) Afib: atrial fibrillation; PVT: portal vein thrombosis; CVA: cerebrovascular accident; DVT: deep vein thrombosis; BID: twice daily; DOAC: direct oral anticoagulant

Patient	Child-Pugh Class	Direct oral anticoagulant utilized	Indication	Dose	Bleeding event	Venous thromboembolism event
1	C	Apixaban	Afib	5 mg BID	No	No
2	C	Apixaban	PVT	2.5 mg BID	Yes (History of hematemesis from variceal bleeding)	Yes (Mesenteric vein thrombosis during encounter)
3	C	Apixaban	PVT	5 mg BID	No	No
4	C	Apixaban	Embolic CVA	5 mg BID	No	No
5	C	Apixaban	DVT	2.5 mg BID	Yes (Fatal variceal hemorrhage in readmission)	No
6	C	Apixaban	History of PVT	5 mg BID	No	No
7	C	Apixaban	Afib	5 mg BID	No	No
8	C	Apixaban	Afib	5 mg BID	No	No
9	C	Apixaban	PVT	5 mg BID	No	No
10	B	Rivaroxaban	History of DVT	20 mg daily	No	No
11	B	Rivaroxaban	Afib	20 mg daily	No	No
12	B	Rivaroxaban	DVT	20 mg daily	No	No

The incidence of thrombotic events in patients that received a DOAC outside of FDA package insert recommendations was 8.3% (n=1/12). The incidence of bleeding events in this subpopulation was 16.6% (n=2/12). Overall, in cirrhotic patients placed on DOACs in this evaluation, the rate of thrombotic events was 7.3% (n=3/41) and the rate of bleeding events was 4.8% (n=2/41). Safety and efficacy outcomes are further categorized in Table 3.

**Table 3:** Safety and efficacy outcomes DOAC: direct oral anticoagulant

Outcome	Incidence, % (n)	DOAC used	
Bleeding prior to encounter	2.4 (1/41)	Apixaban	
Thrombosis prior to encounter	0 (0/41)	-	
Bleeding event during encounter	0 (0/41)	-	
Thrombus during encounter	2.4 (1/41)	Apixaban	
Readmission due to bleed	2.4 (1/41)	Apixaban	
Readmission due to clot	4.9 (2/41)	Rivaroxaban, Apixaban	

Bleeding prior to encounter (n=1/41)

Patient 2 (Table 2): This patient had Child-Pugh Class C cirrhosis, who was admitted for hepatic encephalopathy, and restarted on home apixaban 2.5 mg twice daily (BID) for portal vein thrombosis diagnosed six months ago. The apixaban dose had been reduced from 5 mg BID after one month of initiation of the drug secondary to hematemesis. Per chart and external medication fill history, the patient seemed to be adherent with apixaban 2.5 mg BID.

Thrombosis during encounter (n=1/41) 

Patient 2 (Table 2): On hospital day one, the patient was found to have a new superior mesenteric vein thrombosis and was placed on apixaban 10 mg BID for seven days and discharged on apixaban 5 mg BID. After two months, due to worsening ascites, the patient was admitted for a transjugular intrahepatic portosystemic shunt procedure, when a computerized tomography (CT) scan of the abdomen showed worsening occlusion of the superior mesenteric vein due to thrombus. The patient was initiated on therapeutic enoxaparin and discharged on this regimen.

Readmission due to bleed (n=1/41)

Patient 5 (Table 2): This patient had Child-Pugh Class C admitted for hypotension post hemodialysis and was found to have deep vein thrombosis (DVT) of the right internal jugular vein. The patient was initiated on a heparin drip and remained on this for seven days, before being transitioned to apixaban 2.5 mg BID. Per chart review, the reduced dose for indication of new DVT was thought to be due to renal dose adjustment as the patient was on hemodialysis. The patient was discharged on this reduced dose of apixaban 2.5 mg BID. One month later the patient was re-admitted for hypoglycemia. In this admission, he had an episode of large volume hematemesis from bleeding esophageal varices, which were banded. Further workup was not obtained as unfortunately, the patient died, and the cause of death was deemed to be hemorrhagic shock.

Readmission due to clot (n=2/41)

Patient A

This patient had Child-Pugh Class A cirrhosis, was admitted with pulmonary embolism, and was initiated on rivaroxaban. He was discharged with a prescription to complete the appropriate loading dose, and then initiate a maintenance dose of rivaroxaban 20 mg daily. Five months later, the patient was admitted for bilateral lower extremity swelling and shortness of breath. Imaging showed a large pulmonary embolus with right heart strain, with new DVTs in bilateral lower limbs. The patient was initiated on a heparin drip initially and subsequently discharged on warfarin.

*Patient B* 

This patient had Child-Pugh Class B cirrhosis, was admitted for necrotizing pancreatitis, and was found to have a supratherapeutic INR. The patient was on warfarin at home for a history of DVT and the decision was made to transition the patient to apixaban. The patient was discharged on apixaban 5 mg BID. After six months, the patient was admitted for left lower extremity tenderness and swelling and was found to have a new DVT. The patient reported non-compliance with apixaban, and he had gone two weeks without taking it. The decision was made to re-initiate apixaban as this was not deemed a failure of therapy, and he was loaded with apixaban 10 mg BID for seven days and then 5 mg BID thereafter.

## Discussion

Patients with cirrhosis are at an increased risk of clinically significant bleeding; however, procoagulant and anticoagulant imbalances may also place them at an increased risk of thrombosis. The pro-hemorrhagic and pro-thrombotic changes in patients with cirrhosis are primarily related to the severity of the liver disease [3]. 

As explained further below, according to the manufacturers of both FXa inhibitors, apixaban and rivaroxaban can be used in mild liver disease (Child-Pugh A) if there is no clinically concerning bleeding risk. Only apixaban can be administered in patients with moderate liver disease (Child-Pugh B), while those with severe liver disease (Child-Pugh C) should avoid both agents [4].

Approximately 25% of apixaban is metabolized in the liver, predominately via the cytochrome P450 system, to inactive metabolites; approximately 30% of the drug is renally eliminated, while the remaining is excreted in the stool [5]. Pharmacokinetic studies have shown that Child-Pugh Class A and Child-Pugh Class B cirrhosis has no appreciable effect on the pharmacokinetics of apixaban, and, thus, adjustment of the dose may not be required [6]. Product labeling for apixaban does not provide any dose adjustments in Child-Pugh Class B and states that one should use apixaban with caution in these patients due to the limited availability of clinical and research data. For Child-Pugh Class C, the product labeling does not recommend using apixaban [7].

Approximately 50% of rivaroxaban dose is metabolized via hepatic biotransformation through the cytochrome P450 system. Rivaroxaban is excreted primarily by the kidneys and liver (66% and 28%, respectively) [8]. Usually, in patients with Child-Pugh Class A hepatic impairment, there are minor changes in pharmacokinetic properties compared to healthy individuals. However, in patients with Child-Pugh Class B hepatic impairment, there is an increase in serum rivaroxaban concentration and elimination time, compared to healthy subjects [9]. Product labeling for rivaroxaban does not provide dose adjustment for patients with Child-Pugh Class A cirrhosis, with the limited data available on pharmacokinetic and pharmacodynamic response being similar to healthy subjects. However, it does state that the use should be avoided for patients with Child-Pugh Class B or C cirrhosis [8]. Furthermore, when compared to warfarin, rivaroxaban was found to have a higher rate of major bleeding from the gastrointestinal tract, which may make providers hesitant to use rivaroxaban in this patient population [10].

However, one should be aware that these recommendations are based on relatively scarce data; with each agent typically having very few studies with small sample sizes [4]. Additionally, a study by Potze et al. demonstrated that in patients with decompensated cirrhosis (Child-Pugh Class B and Class C), apixaban and rivaroxaban showed a significantly lower in vitro anticoagulant potency [11]. Despite paucity in clinical data and in vitro studies demonstrating clinical unpredictability of apixaban and rivaroxaban, DOACs have been used by clinicians over low molecular weight heparin or vitamin K antagonists in moderate to severe cirrhotic patients owing to the ease of oral administration and decreased monitoring.

At our hospital, between April 2016 through June 2020, 41 patients with cirrhosis were placed on a DOAC (apixaban or rivaroxaban). Of these, 29.3% (n=12/41) were placed on a DOAC outside of the FDA prescribing recommendations from the respective product labeling, based on Child-Pugh classification. The incidence of the clinical safety events (bleeding events) in this subpopulation was 16.6% (n=2/12), of which one patient, on long-term hemodialysis, had a major bleeding event resulting in a fatal outcome. The other patient had a moderate bleeding event, requiring a dose reduction of apixaban. The incidence of the clinical efficacy events (thrombotic events) in this subpopulation was 8.3% (n=1/12), however, this patient had been previously dose reduced (apixaban 2.5 mg BID) for a prior bleeding event.

A recent retrospective study by Oldham et al. evaluated patients with Child-Pugh B and Child-Pugh C cirrhosis who received either a DOAC (apixaban, rivaroxaban, dabigatran, or edoxaban), enoxaparin, or warfarin [12]. The DOAC population (n=69/101 of which 83% were on apixaban, 14% on rivaroxaban, and 3% on dabigatran) revealed that 31% of patients with Child-Pugh B cirrhosis experienced a bleeding event and 70% of Child-Pugh C cirrhosis patients had a bleeding event. Of the patients in the DOAC group, 4% experienced a thromboembolic event. This was the largest study comparing the use of DOACs with traditional anticoagulation therapy in patients with Child-Pugh B and Child-Pugh C cirrhosis. In the same study, the incidence of bleeding events was 36% in the DOAC group and 22% in the traditional anticoagulation group (p=0.149). Thromboembolic events were reported in 4% of the DOAC group and none in the traditional anticoagulation group (p=0.55). Whereas in our study, the overall rate of bleeding events was 4.8% (n=2/41) and the rate of VTE events was 7.3% (n=3/41) in cirrhotic patients taking DOACs.

Few other studies evaluated the use of DOAC in patients with cirrhosis that also included Child-Pugh classifications; however, these studies mainly included patients with Child-Pugh A hepatic cirrhosis [13-15]. In a study by Hum et al. which included 27 cirrhotic patients receiving DOAC and 18 receiving warfarin or low molecular weight heparin, both groups had similar total bleeding events (8 DOAC vs. 10 other, p=0.12). Recurrent thrombosis occurred in one patient (4%) who received a DOAC and one patient (6%) who received another anticoagulant (p=1.0). There were 17 patients on rivaroxaban and 10 patients on apixaban in this study. Child-Pugh class A (n=11) and B (n=12) patients made up the majority of DOAC patients in this study, with no Child-Pugh class C patients [13]. Another study included 42 patients who took a DOAC and 37 patients who took warfarin. The rate of all-cause bleeding was 16.7% (n=7) in the DOAC group compared to 21.6% (n=8) in the warfarin group (p=0.7). The DOAC group had a rate of failed efficacy of 7.1% (n=3) compared to 8.1% (n=3) in the warfarin group (p=0.9). In the DOAC group, 29 patients took apixaban, nine patients took rivaroxaban, and four patients were on dabigatran. There were 34 Child-Pugh Class A patients in the DOAC group, eight Child-Pugh Class B patients, and no Child-Pugh Class C patients [14]. In a retrospective cohort study of patients with chronic liver disease and atrial fibrillation initiated on oral anticoagulants, all-cause bleeding rates were similar between the groups, with 8.4% (n=75) in the DOAC group and 8.8% (n=158) in the warfarin group (hazard ratio (HR) 0.9, 95%CI 0.4-1.8). 15% (n=11) of the DOAC patients were taking apixaban, 47% (n=35) were taking dabigatran, and 39% (n=29) were taking rivaroxaban. The DOAC group included 48 patients in Child-Pugh Class A, 26 patients in Child-Pugh Class B, and one patient in Child-Pugh Class C [15]. In a nutshell, none of the studies mentioned above demonstrated a statistically significant difference between the use of a DOAC or a traditional anticoagulant with respect to the occurrence of bleeding or thrombotic events. However, given the small sample size and a higher proportion of patients with compensated cirrhosis in these studies, it is difficult to draw conclusions about the safety and efficacy profile of DOACs compared to traditional anticoagulation in patients with moderate to advanced cirrhosis.

As mentioned above, the incidence of thrombotic events in patients placed on a DOAC outside of prescribing recommendations in our study was 8.3%, and the risk of bleeding events was 16.6%. These results were comparable to the Apixaban for the Initial Management of Pulmonary Embolism and Deep-Vein Thrombosis as First-Line Therapy (AMPLIFY) trial comparing the efficacy and safety profile of apixaban with enoxaparin/warfarin therapy in acute venous thromboembolism, in which 2.3% of the patients had thrombotic events and 15% had bleeding events [16]. Our study adds to the small body of evidence evaluating the use of DOAC therapy in patients with Child-Pugh Class B and Class C hepatic cirrhosis. However, it is important to note that this study is subject to limitations. This study relied on retrospective chart review, so some bleeding and/or thrombosis events may have not been documented or patients may not have been graded as carefully by the treating physician as compared to patients in clinical trials. Further, our study had a relatively small sample size. Additionally, this study also only evaluated the use of two DOACs, apixaban and rivaroxaban, with no patients receiving dabigatran or edoxaban.

## Conclusions

The ability to use DOACs in severe liver disease, including Child-Pugh C Class cirrhosis, is appealing as monitoring and/or administration may be difficult with traditional anticoagulants like vitamin K antagonists (warfarin) and low-molecular-weight heparins. Further studies are needed to better understand the safety and efficacy profile of DOACs in this patient population, especially in patients with Child-Pugh Class B and Class C hepatic cirrhosis.
